# Single-Channel Multiple-Receiver Sound Source Localization System with Homomorphic Deconvolution and Linear Regression

**DOI:** 10.3390/s21030760

**Published:** 2021-01-23

**Authors:** Yeonseok Park, Anthony Choi, Keonwook Kim

**Affiliations:** 1 Division of Electronics & Electrical Engineering, Dongguk University-Seoul, Seoul 04620, Korea; dustjrdk@dongguk.edu; 2 Department of Electrical & Computer Engineering, Mercer University, 1501 Mercer University Drive, Macon, GA 31207, USA; choi_ta@mercer.edu

**Keywords:** linear regression, sound source localization, single channel, time of flight, angle of arrival, homomorphic deconvolution, cepstrum, machine learning, Yule–Walker, Prony, Steiglitz–McBride, vehicle

## Abstract

The conventional sound source localization systems require the significant complexity because of multiple synchronized analog-to-digital conversion channels as well as the scalable algorithms. This paper proposes a single-channel sound localization system for transport with multiple receivers. The individual receivers are connected by the single analog microphone network which provides the superimposed signal over simple connectivity based on asynchronized analog circuit. The proposed system consists of two computational stages as homomorphic deconvolution and machine learning stage. A previous study has verified the performance of time-of-flight estimation by utilizing the non-parametric and parametric homomorphic deconvolution algorithms. This paper employs the linear regression with supervised learning for angle-of-arrival prediction. Among the circular configurations of receiver positions, the optimal location is selected for three-receiver structure based on the extensive simulations. The non-parametric method presents the consistent performance and Yule–Walker parametric algorithm indicates the least accuracy. The Steiglitz–McBride parametric algorithm delivers the best predictions with reduced model order as well as other parameter values. The experiments in the anechoic chamber demonstrate the accurate predictions in proper ensemble length and model order.

## 1. Introduction

The signal propagation over the airborne space contains the spatial information of delivery. The sound source localization (SSL) system interprets the received signal to estimate the angle of arrival (AoA) for the signal source. Understanding the spatial information usually requires the extensive processing over the multi-channel signals. The dominant methods utilize the phase differences between the receivers for beamforming [1,2] which can be employed for various applications such as underwater warfare systems. Along with processing power, the number of receivers determines the beamforming performance equivalent to the AoA estimation resolution. The beamforming limitations are confronted with the biomimetics methods. Certain animals including humans can precisely localize sound sources in three-dimensional (3D) space by using the binaural correlation and structure profile [3]. Numerous monaural [4] and binaural [5] sound localization systems have been suggested to mimic the human-like hearing system. Recently, researchers are performing studies to comprehend the propagation on the practical structure with single or dual receivers for precise and feasible SSL systems [6,7,8,9,10,11,12,13,14].

The acoustic information can be used for improving the safety of future autonomous transport systems. The acoustic reception with a single channel can classify the sound source to identify the obstacles and hazards. Beyond the identification, the SSL system provides the further information on the sound source in conjunction with a vision system. The sound propagates the information over the non-line-of-sight (NLOS) locations via diffraction property; hence, the system safety is significantly enhanced by the sound identification and localization on the extended ranges and directions. The system may recognize the direct or indirect endangerment to activate the pre-emptive safety devices which reduce or remove the impact of the imminent collisions. Therefore, the sound is the complementary to vision for navigating mobile objects. This paper extends the SSL methods toward the novel, feasible, and deployable structure to realize them on mobile systems such as vehicles, robots, etc. Observe that the intricate shape of the mobile system produces the complex propagation pathway which requires the complex mathematical model in the SSL algorithms. The proposed SSL system should contain the high scalability and low complexity to meet the objective. The goal of this paper is that the SSL designer can place the receivers on anywhere in the installation system without concern about the hardware and algorithm complexity for optimal performance.

The SSL system for mobile vehicle requires the additional hardware and algorithm complexity compared to the simple sound classifier. The beamforming, monaural, and binaural approach can be used for the SSL system. Note that the arrival level- and time-based methods are not considered for SSL system due to the substantial performance limitations. The beamforming for SSL system provides the accurate localization once the acoustic propagation model is precisely built [2]. The localization resolution is proportional to the receiver number; therefore, the decent number of microphones demonstrate the pinpoint accuracy for AoA finding. In contrast, the beamforming method requires the significant computational power to realize the algorithm with numerous receivers [15]. The non-flat surface of the SSL system creates the extra difficulty to derive the sound propagation model which is the essential components for beamforming realization. The system-wide synchronized sampling of individual signal is necessary to employ the phase information between the receivers. Occasionally, the synchronized sampling causes the realization problem for distributed system in centralized beamforming processing [16].

The monaural and binaural SSL system utilizes the inter-aural level and time differences with frequency variation induced by receiver structure [4,17]. The structure related transfer function generates the variety variation on magnitude, time, and frequency of received signals. The isotropic single or dual receiver without the structure cannot implement the monaural or binaural SSL system due to the absence of variation over the AoA. The head and pinna shape can be adopted as the structure for the SSL system [18,19]. The precise transfer function for the structure exceeds the conventional SSL performance via using the structure produced information [3]. The low number of receivers present the low-profile SSL system as well. However, the structure for the monaural and binaural SSL system may provide the installation limitations on aerodynamic or artistic figure. The placement of the SSL system requires the transfer function analysis again due to the mutual relation by attachment. No explicit parameters are observed for performance scalability in the monaural and binaural SSL system which is not suitable for the super-resolution localization. Numerous research articles [6,7,8,9,10,11,12,13,14] have proposed the improvement methods for a monaural and binaural SSL system by employing the novel algorithm and combining approach; however, the scalability is barely observed without the saturation.

**Figure 1:**
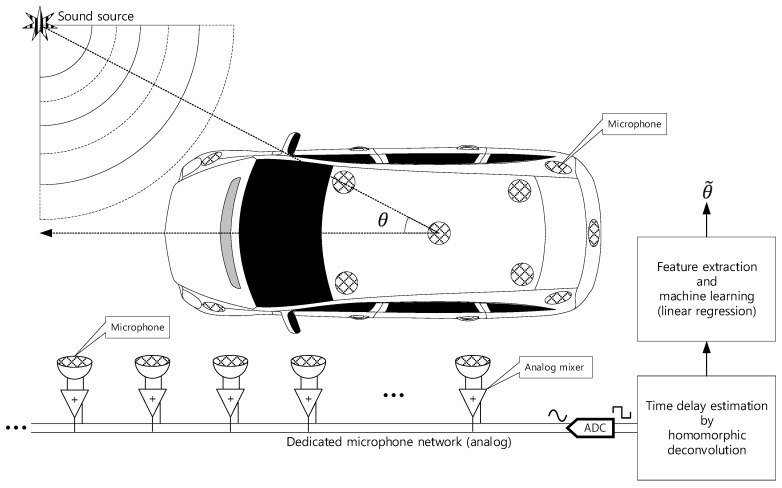
Functional diagram of overall SSL system for transport: θ is the real arrival angle for sound and $\tilde{\theta }$ is the estimated angle. The vehicle shape is illustrated by Nichkov Alexey [22].

This paper proposes the single analog-to-digital converter (ADC) channel SSL system for transport with multiple receivers. The proposed SSL system does not involve any physical structure for localization and the in-situ receivers are placed within the target system. The individual receivers are connected by the analog microphone network which provides the simple communication and connectivity based on asynchronized analog adder circuit [20]. On the microphone network bus, the various time delay data on the mixed signal represents the time of flight (ToF) between microphones. The homomorphic deconvolution (HD) algorithm estimates the implicit ToF distribution based on the homomorphic system. The HD algorithm can produce the output in terms of non-parametric and parametric configurations for the feature extraction which is used for machine learning to derive the AoA. The distribution of the non-parametric method as well as coefficients of the parametric model are important clues to compute AoA information. The proposed vehicle SSL system is extensive to present the complete proposition in single article. Hence, the previous paper [21] demonstrated the ToF estimation for two microphones with non-parametric and parametric HD algorithms. Observe that Figure 1 is parallel to the previous article illustration except the receiver number and machine learning stage. This article describes the SSL system with machine learning based on the findings in previous paper.

The proposed single-channel multiple-receiver (SCMR) SSL system consists of two computational stages as homomorphic deconvolution and machine learning stage indicated in Figure 1. The previous paper verified the performance of ToF estimation by utilizing the non-parametric and parametric HD algorithms [21]. The machine learning trains the algorithm to recognize the AoA from the output of HD algorithms. This paper employs the linear regression with supervised learning for AoA prediction. The SCMR SSL system demonstrates the consistent computational requirement for any problem size in terms of receiver number. The single channel HD computation shows the constant computational burden for any receiver configuration. The extracted features (in non-parametric or parametric distribution) from HD are delivered to the linear regression which establish the uniform computational order for fixed regression degree. Once the receiver configuration presents the proper ToF between microphones to distinguish the AoA distribution, the linear regression estimates the arrival angle accurately with constant computational requirement. The surface and numerical profile of receiver installation hardly affect the SCMR SSL performance when the linear regression is trained deeply with extensive featured dataset. The mathematical model of the sound propagation is not mandatory for SCMR SSL system for 2D or 3D surface, and extensive or reduced number of receivers. However, the dense and considerable dataset should follow the massive computational power for learning process in linear regression.

Recently numerous investigations have been conducted for SSL systems with machine/deep learning and are organized as below. Sun et al. [23] suggests the indoor SSL system based on the generalized cross correlation feature and probabilistic neural network to tolerate the high reverberation and low signal-to-noise ratio (SNR) situation. The convolutional recurrent neural network is developed for joint sound event localization and detection of multiple overlapping sound events in 3D space from a sequence of consecutive spectrogram time frames [24]. Ma, Gonzalez, and Brown present the novel binaural sound localization that combines the model-based information about the spectral characteristics of sound sources and deep neural networks [25]. In underwater ocean waveguides, the generalized regression neural network localizes the sound source based on the normalized sample covariance matrix of input signal [26]. The AoA estimation is performed by the input inter-channel phase differences from the deep neural network-based phase difference enhancement [27]. In the mismatched head-related transfer function condition, the data-efficient and clustering method based on deep neural network is provided to improve binaural localization performance [28].

This paper accomplishes the goals proposed by the authors’ previous SSL publications. The ToFs between microphones were estimated by non-parametric and parametric homomorphic deconvolutions which are served as the fundamental ground for this paper [21]. The asymmetric horizontal pyramidal horns were organized for the far-field monaural SSL system by utilizing cepstral parameters induced by fundamental frequencies [13]. The asymmetric vertical cylindrical pipes around a single microphone realized the small-profile near-field monaural SSL system [12]. To be estimated by homomorphic deconvolution, the direction-wise time delays from the multiple plate structure were designed for the reflective monaural SSL system [11]. The binaural system detected the azimuthal movement of the sound source [10] and the low-power acoustic sensor network localized the target in the distributed field [29] as the efforts for the SSL subject by the authors. Observe that the identical anechoic chamber [30] has been operated for the acoustic experiments and evaluations of the prior works. 

## 2. Non-Parametric and Parametric Homomorphic Deconvolution

**Figure 2:**
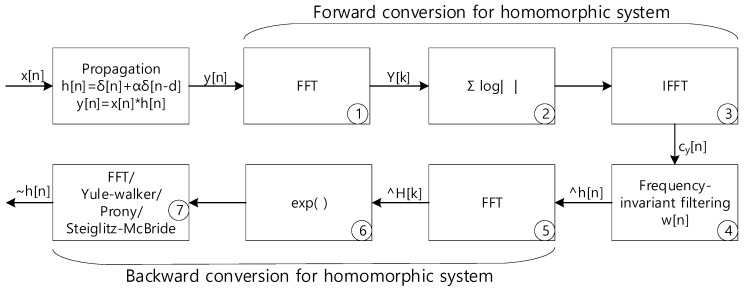
Computational procedure for parametric and non-parametric homomorphic deconvolution.

This section is provided by editing and modifying the previous paper [21] to improve readability of the overall presentation. For further information, the readers are recommended to pursue the article for discovering the detail description on non-parametric and parameter HD. In the paper, the HD utilizes the homomorphic systems in cascade to derive the propagation function which represents ToFs between the receivers. The real cepstrum of the received signal as the geometric series form realizes the forward conversion of the homomorphic system. The robust method to extract the propagation function from the received signal is delivered by the distinct geometric series rates in cepstrum domain. The actual separation in the cepstrum domain is performed by the simple window known as frequency-invariant linear filtering (FILF). Finally, the propagation function for ToF is derived by the backward conversion of the homomorphic system in inverse cepstrum. For non-parametric estimation, the discrete Fourier transform (DFT) or fast Fourier transform (FFT) is extensively used for the real cepstrum. The propagation function model implements the parametric ToF estimation in last stage of HD via applying Yule–Walker [31,32], Prony [32,33], and Steiglitz–McBride [32,34]. Observe that the parametric method calculates model coefficients and the non-parametric technique generates the numerical distribution. Figure 2 illustrates the overall computational procedure for non-parametric and parametric HD algorithm. 

**Figure 3:**
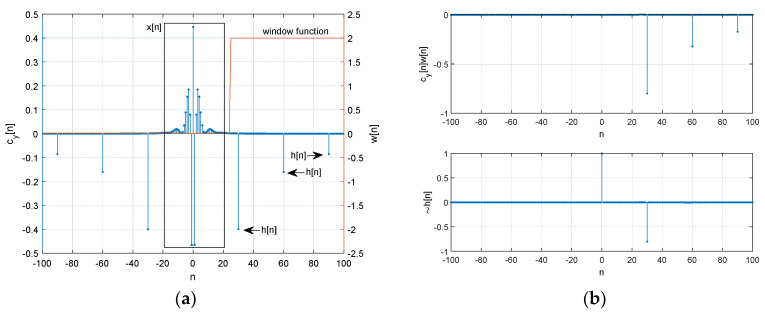
Numerical examples for non-parametric homomorphic deconvolution: (**a**) $c_{y}\left[n\right]$ after forward conversion (x[n] and h[n] areas are indicated); (**b**) windowed $c_{y}\left[n\right]$ in upper and estimated $\tilde{h}\left[n\right]$ in lower. $\tilde{h}\left[n\right]$ is represented by ~h[n].

Figure 3 demonstrates the estimation procedure for two receivers with numerical examples. Both microphones receive the signal x[n] with $dT_{s}$ time difference. The corresponding propagation function h[n] is represented by δ[n]+αδ[n−d] with sampling period $T_{s}$, Kronecker delta function δ[n], and attenuation rate α. The received signal y[n] is the convolution sum as x[n]∗h[n]. The first FFT (stage ① in Figure 2) and IFFT (stage ③) pair with absolute logarithm (stage ②) presents the real cepstrum $c_{y}\left[n\right]$ to divide the signal and propagation distribution. Figure 3a shows the numerical example of forward conversion with marked areas for contributions. The window function w[n] extracts the propagation function. The other FFT (stage ⑤) and IFFT (stage ⑦) pair with exponential function (stage ⑥) reestablishes and estimates the non-parametric propagation impulse response $\tilde{h}\left[n\right]$. Figure 3b presents the example for windowed $c_{y}\left[n\right]$ in upper and derived $\tilde{h}\left[n\right]$ in lower. The original h[n] is δ[n]−0.8δ[n−30] which is identical to the estimated h[n] as $\tilde{h}\left[n\right]$. 

The regressive model is utilized for the parametric estimation to define the peaky distribution in propagation function h[n]. The Yule–Walker, Prony, and Steiglitz–McBride method are devised to provide the signal spectral property. The conventional regressive signal models in time and *z* domain are below.

(1) $$y\left[n\right]+a_{1}y\left[n-1\right]+\cdots +a_{N}y\left[n-N\right]=b_{0}x\left[n\right]+b_{1}x\left[n-1\right]+\cdots +b_{M}x\left[n-M\right]$$

(2) $$H\left(z\right)=\frac{b_{0}+b_{1}z^{-1}+\cdots +b_{M}z^{-M}}{1+a_{1}z^{-1}+\cdots +a_{N}z^{-N}}$$

The Yule–Walker algorithm estimates the model parameters below to describe the propagation function h[n] of a given time signal.

$$\left\{{\tilde{a}}_{1},{\tilde{a}}_{2}\ldots ,\;{\tilde{a}}_{N}\right\}\in \mathbb{C}\;\mathrm{and}\;b_{0}={\tilde{\sigma }}^{2},\;b_{1}=b_{2}=\cdots =b_{M}=0$$

The Prony and Steiglitz–McBride algorithm computes the model coefficients below to represent h[n].

$$\left\{{\tilde{a}}_{1},\ldots ,\;{\tilde{a}}_{N},\;{\tilde{b}}_{0},\;\ldots ,\;{\tilde{b}}_{M}\right\}\in \mathbb{C}$$

The last computational stage (⑦ in Figure 2) of the non-parametric and parametric HD algorithms performs the inverse transformation from frequency to time domain. The Yule–Walker, Prony, and Steiglitz–McBride algorithms are originally devised for forward transformation from time to frequency domain. The conjugated frequency domain distribution to the Yule–Walker, Prony, and Steiglitz–McBride algorithms generate the conjugated parameters for estimation according to Equation (3). X[k] and x[n] is the frequency and time domain data, respectively with FFT in L length.

(3) $${\left(x\left[n\right]\right)}^{*}=\frac{1}{L}{\mathrm{FFT}}_{L}\left(X{\left[k\right]}^{*}\right)={\left({\mathrm{IFFT}}_{L}\left(X\left[k\right]\right)\right)}^{*}$$

Between the microphones, the likelihood of ToF information is produced by the numerical distribution of non-parametric HD. Hence, the time positions standing for the maximum values specify the propagation function estimation. The last inverse Fourier transform (⑦ in Figure 2) of non-parametric HD is replaced for the parametric methods by Yule–Walker, Prony, and Steiglitz–McBride. The ToF positions by maximum values from following regressive model is properly indicated by the derived complex number coefficients for the parametric method.

(4) $${\left|\tilde{h}\left[n\right]\right|}^{2}=\frac{{\left|{\tilde{b}}_{0}+{\tilde{b}}_{1}e^{-j\frac{2\pi }{L}n}+\cdots +{\tilde{b}}_{M}e^{-jM\frac{2\pi }{L}n}\right|}^{2}}{{\left|1+{\tilde{a}}_{1}e^{-j\frac{2\pi }{L}n}+\cdots +{\tilde{a}}_{N}e^{-jN\frac{2\pi }{L}n}\right|}^{2}}\;\left\{{\tilde{a}}_{1},\ldots ,\;{\tilde{a}}_{N},\;{\tilde{b}}_{0},\;\ldots ,\;{\tilde{b}}_{M}\right\}\in \mathbb{C};\;0\leq n\leq L-1$$

**Figure 4:**
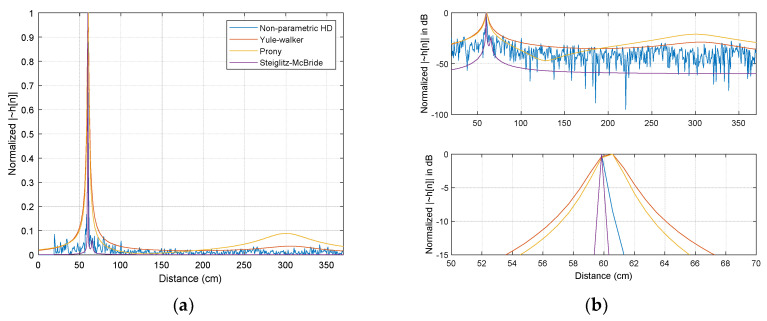
Estimated $\left|\tilde{h}\left[n\right]\right|$ distribution (normalized) with 100 ensemble average length for 60 cm between microphones: (**a**) magnitude distribution; (**b**) decibel distribution in upper and magnified decibel distribution in lower. $\tilde{h}\left[n\right]$ is represented by ~h[n].

The simulations for various SNR and ensemble average length (after logarithm stage in ②) present statistical performance outcomes [21]. The Steiglitz–McBride and non-parametric HD method demonstrate the consistent distribution with low variance and bias in general. The Prony and Yule–Walker methods are subject to the simulation conditions. In both methods, the better statistical performance is produced by the high SNR and long ensemble length overall. The experiments in anechoic chamber similarly provides the equivalent outcomes as the simulation. In the Steiglitz–McBride and non-parametric HD method, near zero bias and variance are shown for the increased ensemble length. The Prony and Yule–Walker method illustrate the performance enhancement in terms of bias and variance for longer ensemble length situations. The FILF window w[n] is determined to remove the 25 sample (18 cm in equivalence) underneath with maximum phase realization in the experiments. Figure 4 indicates the normalized $\left|\tilde{h}\left[n\right]\right|$ with 100 ensemble average length and 60 cm distance situations for non-parametric and parametric HD algorithms.

## 3. Methodology

The SCMR SSL system is realized by the derived the propagation function from non-parametric and parametric HD algorithm. The incident arrival angle determines the ToF distribution for propagation function based on the receiver configuration. To estimate the AoA, the propagation function is properly organized for linear regression. The linear regression generates the predicted output based on the learning process which provides the optimal coefficients from least square procedure. The non-parametric and parametric HD algorithms deliver the unique numerical distribution for propagation function; hence, the proper feature extraction is required for linear regression processing. The following subsections show the feature extraction for data preprocessing and linear regression toward machine learning. 

### 3.1. Feature Extraction

The linear regression is classified as the supervised learning which involves the labeled examples for optimal coefficients. The regression problem is answered by a regression learning algorithm that investigates a collection of labeled examples. Subsequently, the model with optimal coefficients take an unlabeled example for prediction. The feature and label are prepared as below:

(5) $${\left\{\left(x_{i},y_{i}\right)\right\}}_{i=1}^{L}\;\mathrm{where}\;x_{i}\in {\mathbb{R}}^{M\times 1},\;y_{i}\in \mathbb{R}$$

For the *L* dataset, the bold lower case indicates the vector and the lower case represents the scalar. The subscript presents the *i*-th dataset. The feature vector ***x****_i_* corresponds to the label *y_i_*. The individual vector ***x****_i_* is arranged as below.

(6) $$x_{i}={\left[x_{i}^{\left(1\right)}\;x_{i}^{\left(2\right)}\ldots \;x_{i}^{\left(M-1\right)}\;x_{i}^{\left(M\right)}\right]}^{T}$$

The superscript number *n* with parenthesis denotes the *n*-th feature in the vector. Note that there are *M* features in the vector.

(7) $$y_{i}\left[n\right]=x_{i}\left[n\right]*h_{i}\left[n\right]$$

The input *y_i_*[*n*] to the HD algorithm is the convolution sum (denoted by ∗) between sound source signal *x_i_*[*n*] and propagation function *h_i_*[*n*] as shown above for *i*-th iteration. Note that the sound source *x_i_*[*n*] and feature vector ***x****_i_* are individual and independent label each other. The HD algorithm described in Equation (8) specifies the procedure up to ⑤ stage in Figure 2.

(8) $$\hat{H}\left[k\right]=\overset{N-1}{\underset{n=0}{\sum }}\left(\left(\frac{1}{N}\overset{N-1}{\underset{k=0}{\sum }}\frac{1}{E}\overset{E}{\underset{i=0}{\sum }}\left(\log\left|\overset{N-1}{\underset{n=0}{\sum }}y_{i}\left[n\right]e^{-j\frac{2\pi }{N}kn}\right|\right)e^{j\frac{2\pi }{N}kn}\right)w\left[n\right]\right)e^{-j\frac{2\pi }{N}kn}$$

After the $\hat{H}\left[k\right]$ computation in HD algorithm, exponential (⑥ stage) and final Fourier transform (⑦ stage) are necessary to complete non-parametric HD calculation. Below equation provides the final steps for the non-parametric HD algorithm.

(9) $$\overset{˘}{h}\left[n\right]=\frac{1}{N}\overset{N-1}{\underset{k=0}{\sum }}e^{\hat{H}\left[k\right]}e^{j\frac{2\pi }{N}kn}$$

The non-parametric HD is normalized and bisected as below for feature extraction. Observe that first half of the non-parametric HD delivers the propagation function due to the symmetricity of Fourier transform.

(10) $$\tilde{h}\left[n\right]=\frac{\left|\overset{˘}{h}\left[n\right]\right|}{\max\left|\overset{˘}{h}\left[n\right]\right|}\;\mathrm{for}\;0\leq n\leq \frac{N}{2}$$

A certain range of the HD distribution presents the propagation function based on the ToF over the receiver configuration. The lower bound initiates from the window function *w*[*n*] as *W* value. The window function removes the sound source signal *x_i_*[*n*] by using the weight function in frequency-invariant filtering in HD ④ stage of Figure 2. Additionally, the non-parametric HD $\tilde{h}\left[n\right]$ is zero in *W* below. Note that *w*[*n*] is non-zero from *W* + 1 time index as shown in Figure 3a.

The *n_max_* is the maximum time delay caused by the receiver configuration. Once the *d* is the maximum distance between receivers, the *n_max_* can be calculated by $\mathrm{round}\left(df_{s}/c\;\right)$, where *f_s_* is sampling frequency and *c* is sound speed.

(11) $$x_{i}^{\left(l\right)}=\tilde{h}\left[l+W\right]\;1\leq l\leq n_{max}-W$$

**Figure 5:**
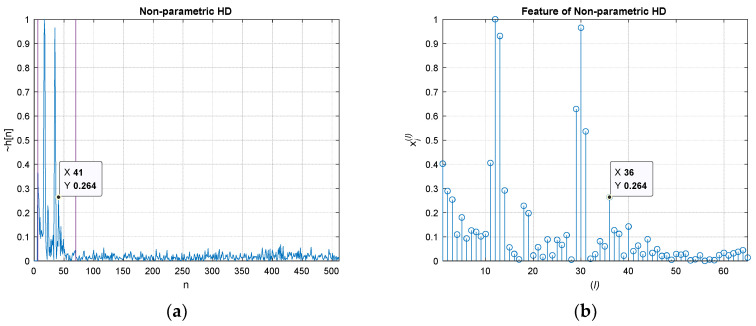
Feature extraction for non-parametric HD by direct feature extraction: (**a**) non-parametric homomorphic deconvolutions (HD) distribution with range indicators (two purple lines) for the feature; (**b**) the extracted feature vector for non-parametric HD. The highlighted values indicate the identical position for the HD value and feature value, respectively. The *x* value is shifted due to the *W* value in window function.

Equation (11) represents the extraction in $\tilde{h}\left[n\right]$ from *W* + 1 to *n_max_* for features as $x_{i}^{\left(l\right)}$. Figure 5 demonstrates the *direct feature extraction* for non-parametric HD. In left figure, the normalized non-parametric HD distribution is illustrated with range indicator (purple vertical lines) which presents the span from *W* + 1 to *n_max_*. On right figure, the extracted feature vector is pictorialized by stem plot. Note that the highlighted values on both figures indicate the identical position and value.

The parametric HD provides the coefficient values for the signal model based on the *Z*-transform as shown in Equation (2). The direct feature extraction, which is applied to the non-parametric HD feature, cannot be employed for parametric HD because of numerical inaccessibility for HD distribution. According to the relationship between *Z*-transform and Fourier transform, the unit circle in *z* domain should correspond to the feature vector for linear regression. The *radial projection* presents the implicit non-linear transform to convert the coefficients for the HD distribution. Equation (12) is the derived coefficients from parametric HD algorithms. Note that the Yule–Walker method only computes the denominator coefficients *a_i_* since the autoregressive model is utilized.

(12) $$\left\{{\tilde{a}}_{1},\ldots ,\;{\tilde{a}}_{P},\;{\tilde{b}}_{0},\;\ldots ,\;{\tilde{b}}_{Q}\right\}=\mathrm{ParametricHD}\left({\left(e^{\hat{H}\left[k\right]}\right)}^{*}\right)\;\left\{{\tilde{a}}_{1},\ldots ,\;{\tilde{a}}_{P},\;{\tilde{b}}_{0},\;\ldots ,\;{\tilde{b}}_{Q}\right\}\in \mathbb{C}$$

The poles of the model are the dominant components to create the peaky response in HD distribution; therefore, the poles are computed and considered as below. Note that the solution of following polynomial represents the poles of the given regressive model.

(13) $$1+{\tilde{a}}_{1}z^{-1}+{\tilde{a}}_{2}z^{-2}+\ldots +{\tilde{a}}_{P}z^{-P}=\left(1-\mu_{1}z^{-1}\right)\left(1-\mu_{2}z^{-1}\right)\ldots \left(1-\mu_{P}z^{-1}\right)$$

The individual pole consists of magnitude and phase as below.

(14) $$\mu_{u}=r_{u}e^{j\theta_{u}}\;\mathrm{for}\;1\leq u\leq P\;j=\sqrt{-1}$$

The phase in *z* domain corresponds to the time delay in HD distribution. Hence, the range of the $\theta_{u}$ is employed for radial projection. Similar to the non-parametric HD, the lower bound initiates from the window function *w*[*n*] as *W* value and the *n_max_* is the maximum time delay caused by the receiver configuration. Equation (15) delivers the conversion from the pole phase to the time delay and the pole magnitude to the feature value.

(15) $$l=\mathrm{round}\left(\frac{\theta_{u}}{\Delta }\right)+1\to x_{i}^{\left(l\right)}=r_{u}\;\mathrm{only}\;\mathrm{for}\;\Delta W\leq \theta_{u}\leq \Delta n_{max}\;\mathrm{where}\;\Delta =\frac{2\pi }{N},\;l\in \mathbb{N}$$

The circular resolution Δ indicates the radian distance for the time delay and denotes the radian per sample. The radian range for propagation function is given as above based on the receiver configuration. The poles within the range are involved for the radial projection which assigns the corresponding magnitude on the specific time delay. The temporal location is calculated by dividing the radian value with the resolution Δ. Observe that the time delay should be rounded to the nearest natural number. Overall, the radial projection matches the unit circle arc in *z* domain with HD distribution for feature extraction. Except the pole locations, the numbers in the feature vector are zeros. Due to the limited order in parametric method, the sparse numerical distribution is expected in feature vector. Note that the feature vector for the non-parametric HD provides the values most likely non-zeros because of direct feature extraction.

**Figure 6:**
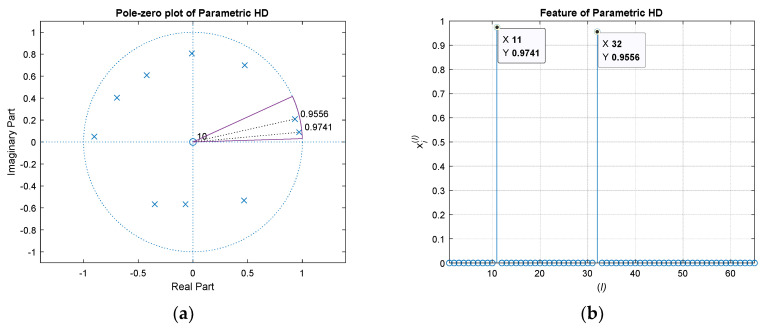
Feature extraction for parametric HD by radial projection. (**a**) Yule–Walker HD (order 10) pole-zero plot with range indicator (purple arc) for feature; (**b**) Extracted feature vector for Yule–Walker HD. The highlighted values indicate the identical positions for Yule–Walker HD pole values and feature values, respectively. The *x* value is derived by circular resolution.

Figure 6 shows the feature extraction for the Yule–Walker parametric HD by radial projection. The pole-zero plot presents the poles and zeros for the order 10 Yule–Walker algorithm model. Observe that the complex number input $e^{\hat{H}\left[k\right]}$ to the parametric method provides the non-symmetric distribution of pole and zero locations. The arc in the pole-zero plot specifies the range for the radial projection. The feature vector in right figure demonstrates the two poles in the range arc. Other than the poles, the values in the feature vector are zeros. The size of feature vector for non-parametric and parametric HD algorithms are identical. The complexity of the extractions is also comparable since one is direct mapping and the other is circular assigning.

### 3.2. Linear Regression

The linear regression [35] predicts the output based on the linear combination of features. The individual feature with coefficient is cumulated for output estimation close to the label for the feature vector. The linearity in the regression model indicates the linearity in terms of coefficients; therefore, the higher order feature polynomial can be used for the regression. The SSL system employs the linear regression model contains an intercept, linear terms, and pair products of distinct features as below.

(16) $$y_{i}=c_{0}+c_{1}x_{i}^{\left(1\right)}+c_{2}x_{i}^{\left(2\right)}+\cdots +c_{M}x_{i}^{\left(M\right)}+c_{M+1}x_{i}^{\left(1\right)}x_{i}^{\left(2\right)}+c_{M+2}x_{i}^{\left(1\right)}x_{i}^{\left(3\right)}+\cdots +c_{R}x_{i}^{\left(M-1\right)}x_{i}^{\left(M\right)}+\epsilon_{i}$$

Note that the $\epsilon_{i}$ is the error or residual between the label and estimation. The regression length except intercept is calculated by adding the feature length *M* with its combination to take two terms as below.

(17) $$R=M+\left(\begin{array}{c} M\\ 2 \end{array}\right)=M+\frac{M!}{\left(M-2\right)!2!}$$

For *L* dataset, the linear regression in matrix form is shown as below.

(18) $$\left[\begin{array}{c} y_{1}\\ y_{1}\\ \vdots \\ y_{L} \end{array}\right]=\left[\begin{array}{cc} \begin{array}{cccc} 1 & x_{1}^{\left(1\right)} & \cdots & x_{1}^{\left(M\right)}\\ 1 & x_{2}^{\left(1\right)} & \cdots & x_{2}^{\left(M\right)}\\ \vdots & \vdots & \ddots & \vdots \\ 1 & x_{L}^{\left(1\right)} & \cdots & x_{L}^{\left(M\right)} \end{array} & \begin{array}{ccc} x_{1}^{\left(1\right)}x_{1}^{\left(2\right)} & \cdots & x_{1}^{\left(M-1\right)}x_{1}^{\left(M\right)}\\ x_{2}^{\left(1\right)}x_{2}^{\left(2\right)} & \cdots & x_{2}^{\left(M-1\right)}x_{2}^{\left(M\right)}\\ \vdots & \ddots & \vdots \\ x_{L}^{\left(1\right)}x_{L}^{\left(2\right)} & \cdots & x_{L}^{\left(M-1\right)}x_{L}^{\left(M\right)} \end{array} \end{array}\right]\left[\begin{array}{c} c_{0}\\ c_{1}\\ \vdots \\ c_{M}\\ c_{M+1}\\ \vdots \\ c_{R} \end{array}\right]+\left[\begin{array}{c} \epsilon_{1}\\ \epsilon_{2}\\ \vdots \\ \epsilon_{L} \end{array}\right]$$

The short representation is presented with proper alphabets as below. Observe that the bold upper case denotes the matrix.

(19) $$y=Xc+\epsilon \;\mathrm{where}\;y\in {\mathbb{R}}^{L\times 1},\;X\in {\mathbb{R}}^{L\times \left(R+1\right)},\;c\in {\mathbb{R}}^{\left(R+1\right)\times 1},\;\epsilon \in {\mathbb{R}}^{L\times 1}$$

The solution of the matrix algebra above can be derived by minimizing the sum of squared residuals. Equivalently, the solution can be introduced from the normal equation [36] which multiply the ***X****^T^* for each side front of the above equation. Below is the solution known as the least square solution.

(20) $$c={\left(X^{T}X\right)}^{-1}X^{T}y$$

In general, the normal equation for least square solution presents the low numerical stability because of high condition number for ***X****^T^**X***. The low stability signifies the high sensitivity to the matrix perturbations in numerical computation. In order to improve the stability, the QR decomposition can be used for the least square solution [37]. The QR decomposition divides the ***X*** into a product of ***QR*** of an orthogonal matrix ***Q*** and an upper triangular matrix ***R*** as below.

(21) $$X=QR\;\mathrm{where}\;Q\in {\mathbb{R}}^{L\times \left(R+1\right)},\;R\in {\mathbb{R}}^{\left(R+1\right)\times \left(R+1\right)}$$

According to the orthonormal property, matrix ***Q****^T^**Q*** is equivalent to the identity matrix ***I***. The matrix ***R*** inversion is existed as long as all diagonal elements of the ***R*** is not zero. The previous least square solution can be written as below by using the QR decomposition.

(22) $$c={\left(X^{T}X\right)}^{-1}X^{T}y={\left(R^{T}Q^{T}QR\right)}^{-1}R^{T}Q^{T}y=R^{-1}{\left(R^{T}\right)}^{-1}R^{T}Q^{T}y\;\mathrm{for}\;\det\left(R\right)=\overset{R+1}{\underset{k=1}{\prod }}r_{kk}\neq 0$$

The least square solution based on the QR decomposition is simplified as below.

(23) $$Rc=Q^{T}y$$

Due to the ***R*** matrix property, upper triangular matrix, the solution to find the ***c*** vector can be quickly solved by back substitution. Various QR decomposition algorithms are available for unique complexities and stabilities [38,39,40].

## 4. Simulations

Prior to discuss the SCMR SSL system simulation, the overall algorithmic procedures are required to summarize for fluent readability. The sound source is propagated over the medium with multiple arrivals on single-channel multiple-receiver configuration. The AoA determines the time delays between the arrivals and establishes the corresponding propagation function *h*[*n*]. The HD algorithms decompose the received signal *y*[*n*] for *h*[*n*] by non-parametric and parametric methods. The features for the linear regression are prepared with direct feature extraction (non-parametric HD) and radial projection (parametric HD). Using the labeled datasets, the linear regression based on the QR decomposition performs the least square solution for optimal coefficients to predict the AoA. The linear regression provides the accurate estimations for unlabeled received signal *y*[*n*].

The first step in simulation is to decide the receiver locations for the proposed SSL system. Note that the SSL system understands the datasets for prediction by calculating the coefficients of the linear model. Unlike the conventional beamforming algorithms [1,2], the SCMR SSL system does not involve the sophisticated mathematical model to place the receivers and to derive the direction. However, the optimal placements should be found by using the brute force method which consists of systematically enumerating all possible receiver configurations for the least prediction error. The extensive simulation datasets are necessary for searching the best receiver positions. The SSL system performance is numerated by root mean square error (RMSE) as below.

(24) $$RMSE\left(\hat{y}\right)=\sqrt{\frac{1}{L}\overset{L}{\underset{i=1}{\sum }}{\left({\hat{y}}_{i}-y_{i}\right)}^{2}}$$

The *y_i_* is the real output (label) of the given input features $x_{i}^{\left(j\right)}$. The estimated output ${\hat{y}}_{i}$ is the output of the linear regression based on the calculated coefficients ${\tilde{c}}_{i}$ in Equation (23).

(25) $${\hat{y}}_{i}={\tilde{c}}_{0}+{\tilde{c}}_{1}x_{i}^{\left(1\right)}+{\tilde{c}}_{2}x_{i}^{\left(2\right)}+\cdots +{\tilde{c}}_{M}x_{i}^{\left(M\right)}+{\tilde{c}}_{M+1}x_{i}^{\left(1\right)}x_{i}^{\left(2\right)}+{\tilde{c}}_{M+2}x_{i}^{\left(1\right)}x_{i}^{\left(3\right)}+\cdots +{\tilde{c}}_{R}x_{i}^{\left(M-1\right)}x_{i}^{\left(M\right)}$$

**Figure 7:**
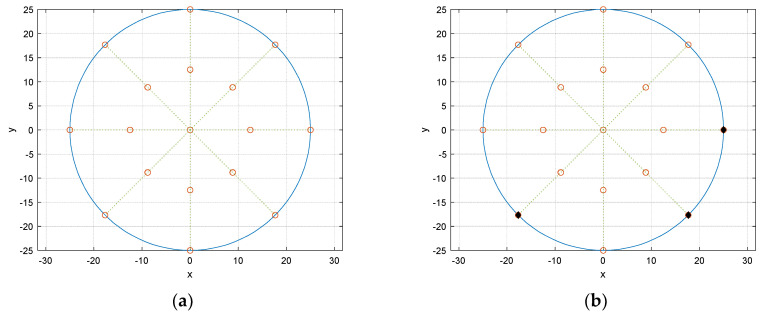
Receiver configuration in centimeter. (**a**) Receiver grid—blank circle indicates the possible receiver locations; (**b**) example configuration—filled circle specifies the occupied location with receiver.

The receiver configuration presents the circular fashion in receiver placement. From the origin, two concentric circles are located with 25 cm and 50 cm diameter, respectively. The circular angle is divided by 8 directions and individual angular distance between the adjacent directions is π/4. The receiver grid is the overlap between the concentric circles and radial directions as shown in Figure 7a which demonstrates the possible receiver locations as blank circles. Figure 7b exhibits the example of the receiver locations with 3 sensors. The number of selections for placing receivers in the given grid can be calculated by simple binomial coefficient. For instance, 17 grid points are available in Figure 7 and 3 receivers are accessible the 680 possibilities for the combination. 

With given receiver locations, the arrival times can be computed by direct distance from the sound source. Additionally, the distance provides the attenuation based on the inverse square law which describes the magnitude of propagation signal over the distance. The equation for inverse square law is shown in below.

(26) $$L_{p}\left(r_{i}\right)=20\log\left[\frac{a}{r_{i}-r_{0}}\right]$$

The *L_p_*(*r_i_*) is the sound pressure level in decibels at the distance *r_i_*. The terms *a* and *r*_0_ are model parameter for amplitude and distance, correspondingly. The acoustic anechoic chamber used in the experiments records the signal with various distance to measure and compute the parameters for precise simulation. The derived values are *a* = 36.1438 and *r*_0_ = 0.0043 cm for the given anechoic chamber [29]. For the sensor configuration, the inverse square law equation calculates the relative magnitude difference between the receivers in numerical simulation. 

The simulation parameters are presented in Table 1. The input data is generated by the numbers such as SNR, angle range, sound speed, receiver count, and etc. The non-parametric and parametric HD are performed by the values for instance ensemble length, frame length, etc. Some parameters show the certain proportionality over the performance; however, the purpose of the pilot simulation is to deliver the optimal receiver configuration by RMSE outcome with fixed parameter values. Further analysis determines the ensemble length and parametric order based on the optimal receiver configuration.

**Figure 8:**
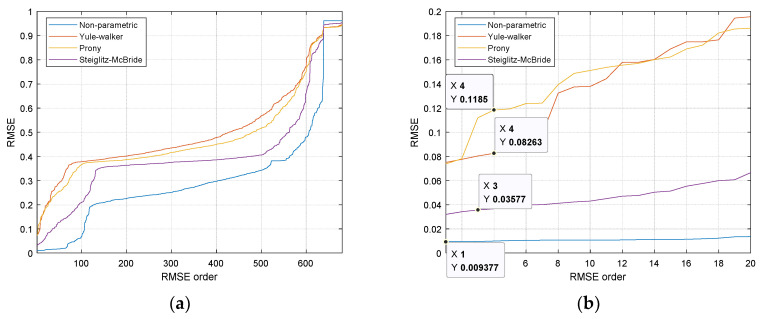
The root means square error (RMSE) performances for all 680 possible receiver configurations. (**a**) Ascending rank order from left to right; (**b**) magnifies the left-bottom corner (highlighted numbers represent the particular receiver configuration).

The RMSE performances for all 680 possible receiver configurations are demonstrated in Figure 8 with ascending order from left to right. The simulation was performed by the 14 computers with intel i7-7700 processor and DDR4 32GB memory for 25-h execution time. The MATLAB parallel processing toolbox provides the coarse-grained parallelism by dividing the for-loop iterations over the multi-core processor. Figure 8b magnifies the left-bottom corner of Figure 8a to select the optimal configuration. The highlighted numbers in each HD algorithms represent the particular receiver configuration which indicates the overall best performance. Note that the *x* numbers in Figure 8b specify the performance rank. The chosen configuration shows the 1, 3, 4, and 4 rank in non-parametric, Steiglitz–McBride, Yule–Walker, and Prony HD, respectively. 

**Figure 9:**
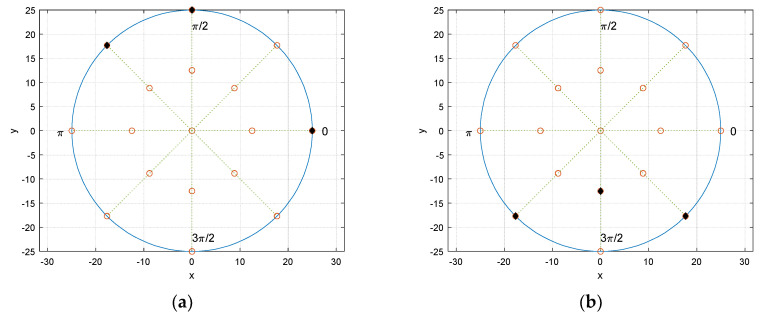
Selected receiver configurations: (**a**) best; (**b**) worst.

The selected receiver configuration is presented in Figure 9a. The black filled dots represent the receiver locations over the potential blank positions. The asymmetricity with wide distribution in locations provides the prominent and distinctive arrival time distribution for linear regression. One of the worst performance configurations is depicted in Figure 9b. Contrary to the best receiver distribution, the configuration demonstrates the symmetricity and tight spreading which delivers the significant ambiguity for linear regression. The higher number of receivers expects the better performance and the three-receiver configuration is the minimum requirement to predict the half circular angle. 

**Figure 10:**
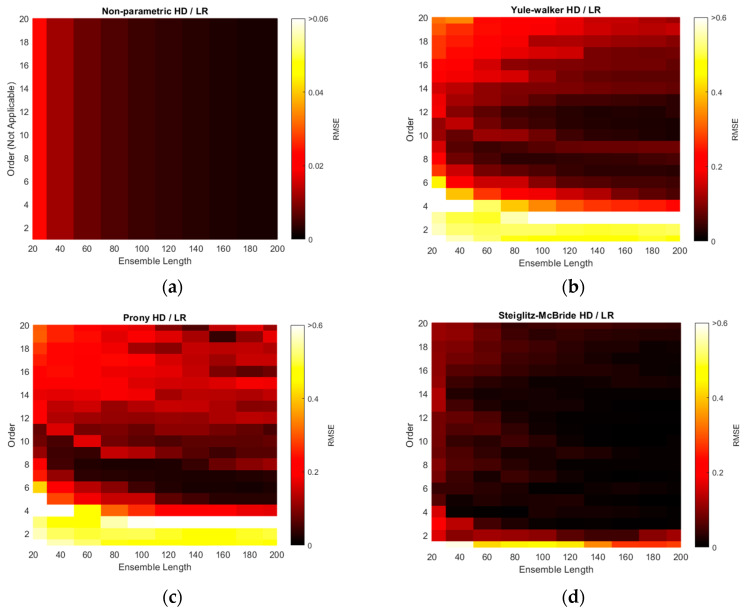
Simulated RMSE distributions of HDs with linear regression (LR) over various ensemble lengths and model orders. Table 1 parameters are applied. (**a**) Non-parametric HD/LR (reduced color scale); (**b**) Yule–Walker HD/LR; (**c**) Prony HD/LR; (**d**) Steiglitz–McBride HD/LR.

The RMSEs of non-parametric and parametric HD with linear regression (LR) are illustrated with various ensemble lengths and model orders in Figure 10. The model order is changed from 1 to 20 in every 1 step and the ensemble length is modified from 20 to 200 in every 20 interval. Note that the non-parametric HD/LR contains the parameter of ensemble length only. The order in the non-parametric HD/LR in Figure 10a is dummy variable to compare with the performance of the other HD/LR algorithms. Additionally, the color scale for non-parametric HD/LR in Figure 10a indicates the reduced range because of the high consistent and low variance RMSE distribution. The longer ensemble length in non-parametric HD/LR presents the daker shade in the RMSE figure on right. The Yule–Walker HD/LR shows the daker lake and river around the order 11/length 160 and order 10 ~ order 7, respectively. The Prony HD/LR demonstrates the darker river similar to the Yule–Walker HD/LR distribution. The Steiglitz–McBride HD/LR displays the darker district above the order 2 and length 160 boundary. The non-parametric HD/LR and Steiglitz–McBride HD/LR establish the best overall performance in Figure 10.

The minimum RMSE values for HD/LR algorithms are organized with corresponding ensemble length and model order in Table 2. In general, the longer ensemble length presents the improved performance in prediction. The parametric HDs with linear regression do not demonstrate the performance proportionality in terms of model order due to the under- and over-determined parameter. The Yule–Walker HD/LR and Prony HD/LR show the similar performance in order 11 and 6, respectively. The Steiglitz–McBride HD/LR indicates around 70 times better performance than the other parametric HDs in order 10. The non-parametric HD/LR RMSE is located between two performance levels. Note that the worst minimum RMSE in Table 2 is 1.2514 degree.

**Figure 11:**
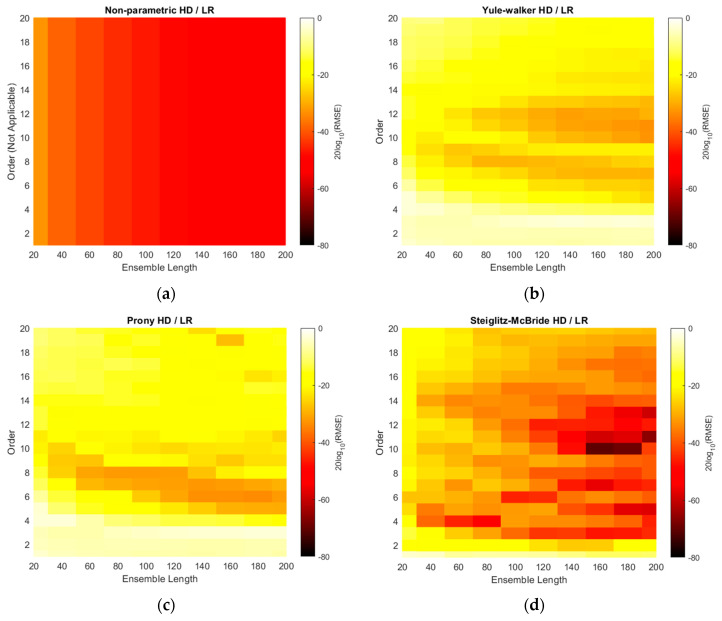
Simulated RMSE distributions in decibel scale for HD/LRs over various ensemble lengths and model orders. Table 1 parameters are applied. (**a**) Non-parametric HD/LR; (**b**) Yule–Walker HD/LR; (**c**) Prony HD/LR; (**d**) Steiglitz–McBride HD/LR.

The RMSE distributions of Figure 10 are recharted in Figure 11 with the decibel scale. Observe that all plots in Figure 11 are coded with single color scale. Hence, the RMSE performances can be sorted as Yule–Walker, Prony, non-parametric, and Steiglitz–McBride HD/LR in ascending order by examining the color shade. The Yule–Walker HD/LR shows the sweet spot around the model order 11 and 12 with ensemble length 120 and above. The Prony HD/LR demonstrates the sweet belt along with model order 6 and 7 for ensemble length 60 and above. The Steiglitz–McBride HD/LR presents the deeper and narrower sweet spot in model order 10 and ensemble length 160. As expected, the non-parametric HD/LR delivers the consistent and proportional performance improvement for longer ensemble length. 

The simulation above is performed to find the optimal receiver locations for three-sensor configuration. Among the radial configuration, the asymmetric and wide distribution is selected over the 680 possibilities. The RMSE is used for evaluation and the error is defined as the difference between the real and predicted angle. Over the optimal receiver configuration, the range of model orders and ensemble lengths were placed to calculate the RMSEs for the non-parametric and parametric HD with linear regression. The RMSE distributions represent that the longer ensemble length and particular model order provide the least RMSE value. The Steiglitz–McBride HD/LR shows the best performance and Yule–Walker HD/LR demonstrates the least accuracy in this simulation.

## 5. Results

The field experiments are realized and evaluated in an anechoic chamber which is verified to show the limited conformance with ISO 3745 [41] for the 1 kHz–16 kHz 1/3 octave band in a hemi-free-field mode and for the 250 Hz–16 kHz 1/3 octave band in a free-field mode [30]. The proposed SCMR SSL methods are evaluated with the free-field mode which involves entirely covered surfaces for all directions with acoustic pyramids. The experiment configuration consists of three microphones with given locations and one speaker with far-field provision. The direction and angle between receivers and transmitter are guided by the line laser (GLL 3–80 P, Bosch, Gerlingen, Germany) placed above the sound source speaker. The far-field provision is maintained by keeping one meter the minimum distance for signal propagation.

**Figure 12:**
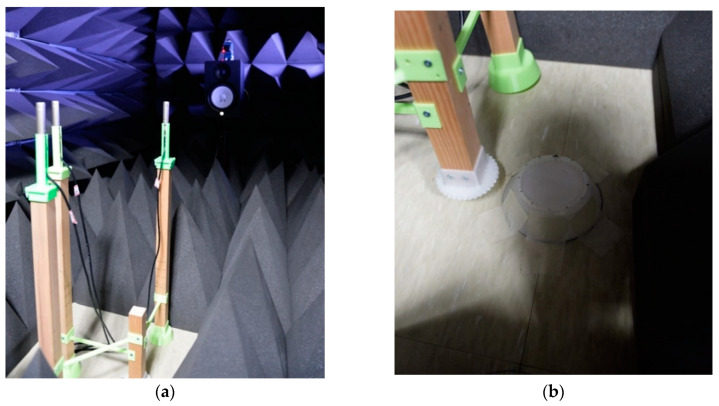
Acoustic experiment in the anechoic chamber: (**a**) three microphones and speaker configuration with laser guidance; (**b**) angular movement engager—the pair of saw-toothed wheels with engraved and intagliated shape for 10° rotation.

The microphones are located on the frames made of lumbers with plastic connectors. The structure sustains the microphone height and receiver shape for angular movements. Observe that the sound source and receiver level should be identical for horizontal propagation. The angle movement for AoA is engaged by the pair of saw-toothed wheels with engraved and intagliated shape for 10° rotation. As shown in Figure 9a, the three receivers are located on the radial pattern with concentric circles; therefore, the center of the circles provides the angular movement center which is dedicated for saw-toothed wheel pair. The overall experiment configuration is illustrated in Figure 12a and the receiver center is shown in Figure 12b for the angular movement engager. The plastic parts are realized by the 3D printer (Replicator 2, MakerBot, Brooklyn, NY, USA) from the polylactic acid (PLA) filament. 

The analog mixer (MX-1020, MPA Tech, Seoul, Korea) connects to the microphones (C-2, Behringer, Tortola, British Virgin Islands) for single channel signal which is digitized by the computer-connected audio device (Quad-Capture, Roland, Hamamatsu, Japan). The speaker (HS80M, Yamaha, Hamamatsu, Japan) is also wired to the audio device to generate the wideband signal. The real-time audio for generation, reception, and execution is processed by the MATLAB system object with the audio stream input/output (ASIO) driver. With 48 kHz sampling rate, the audio is recorded for 20 s in every designated angles. In order to reduce the interruption by transition conditions, the first and last one second data is eliminated. Therefore, the distinct angle experiments employ the overall 18 s of recorded data. Each data frame is established by 1024 samples and the new ensemble average process is begun after the 10 frames later. For data consistency, the rest of the frames are overlapped with given parameter. The experiment parameters are presented in the Table 3. 

**Figure 13:**
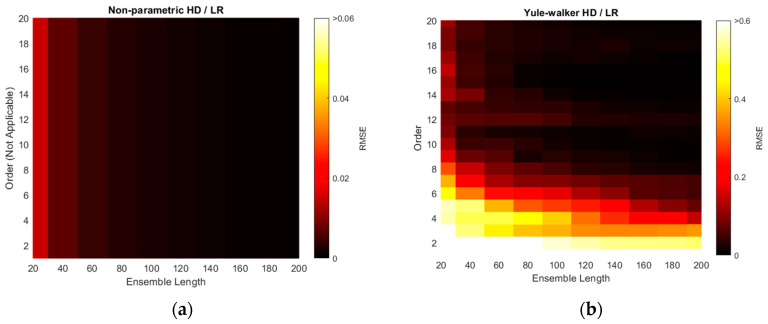

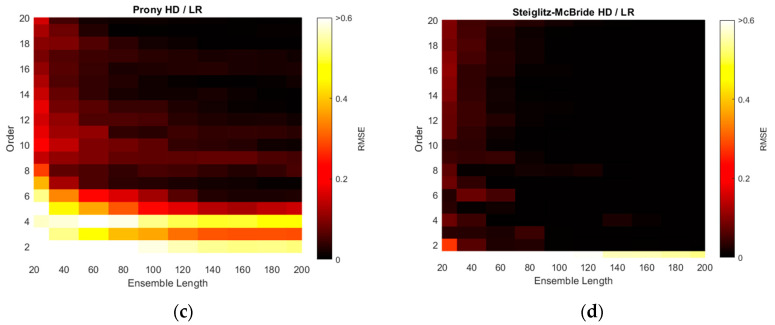
Experimented RMSE distributions of HD/LRs over various ensemble lengths and model orders. Table 3 parameters are applied. (**a**) Non-parametric HD/LR (reduced color scale); (**b**) Yule–Walker HD/LR; (**c**) Prony HD/LR; (**d**) Steiglitz–McBride HD/LR.

Similar to the simulation results, the RMSEs of non-parametric and parametric HD with linear regression are demonstrated with ensemble lengths and model orders in Figure 13. Note that the model order and color scale in the non-parametric HD/LR in Figure 13a is inconsistent with the other RMSE figures as discussed in simulation section. Compare to the simulation Figure 10, the RMSE colors are represented by the darker shade overall which indicates the improved performance in predictions. The longer ensemble length provides the reduced RMSE in all HD/LR algorithms. Additionally, the higher model order delivers the better prediction performance in marginal manner because of the shade gradation in vertical direction. The performance glitches can be observed in order 12 and 9 in Yule–Walker and Prony HD/LR method, respectively. The Steiglitz–McBride HD/LR shows darkest RMSE shade which is below 0.1 radian for most of area except 20 and 40 frames for ensemble length. 

**Figure 14:**
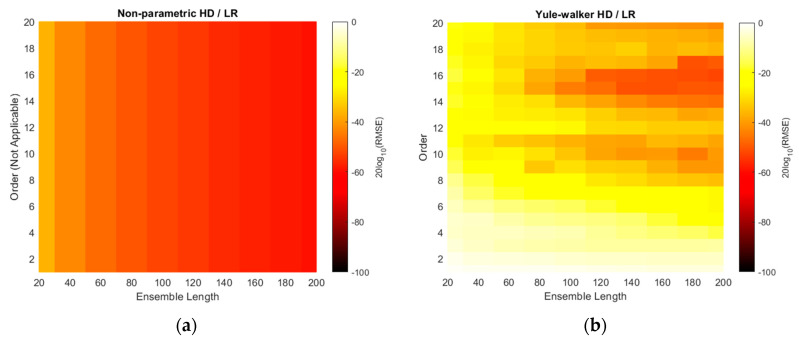

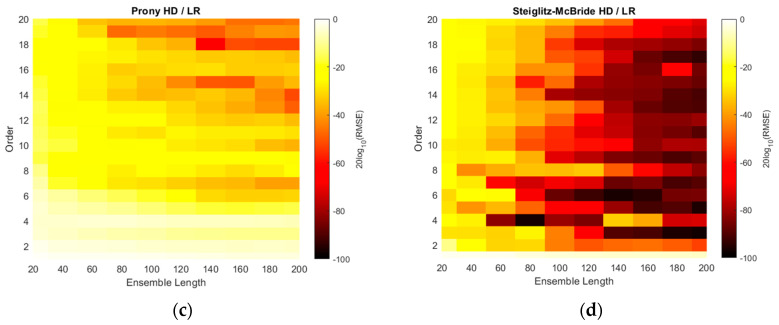
Experimented RMSE distributions in decibel scale for HD/LRs over various ensemble lengths and model orders. Table 3 parameters are applied. (**a**) Non-parametric HD/LR; (**b**) Yule–Walker HD/LR; (**c**) Prony HD/LR; (**d**) Steiglitz–McBride HD/LR.

The RMSE plots are changed to the decibel scale in Figure 14. The consistent and wide scale is used for non-parametric and parametric HD/LRs. Note that the RMSE approaches down to the −100 dB in Steiglitz–McBride HD/LR method. The non-parametric HD/LR presents the coherent and proportional performance enhancement for longer ensemble length. The Yule–Walker and Prony HD/LR show the similar performance with distinct areas and patterns for darker shade. Compare to the simulation result in Figure 11, higher model order produces the lower RMSE overall for Yule–Walker and Prony HD/LR. The Steiglitz–McBride HD/LR method indicates the extensive area for below −60 dB RMSE with uneven boundary of ensemble length 80 above and model order 3 higher. The deep dark shade can be perceived in the low model order as below as 3. 

Table 4 establishes the minimum RMSE values for HD/LR algorithms with corresponding ensemble length and model order. The experiments produce the performance in terms of minimum RMSE with 2, 9, 40, and 21 times better than the simulation counterparts for Table 2 listing order. Therefore, the non-parametric and Steiglitz–McBride HD/LR method show 2 and 21 times lower RMSE than the simulation minimum values, correspondingly. Note that the experiments are performed with identical parameters indicated in simulation specifications except SNR and angle resolution. Overall, the longer ensemble length delivers the higher prediction performance for all HD/LR algorithms. The minimum RMSEs are located in the higher model order than the simulation outcomes for Yule–Walker and Prony HD/LR. The Steiglitz–McBride HD/LR method presents the best performance in low model order 3 since the Steiglitz–McBride algorithm accurately describes the propagation function as shown in previous paper [21]. Note that the worst minimum RMSE in Table 4 is 0.1411 degree.

**Figure 15:**
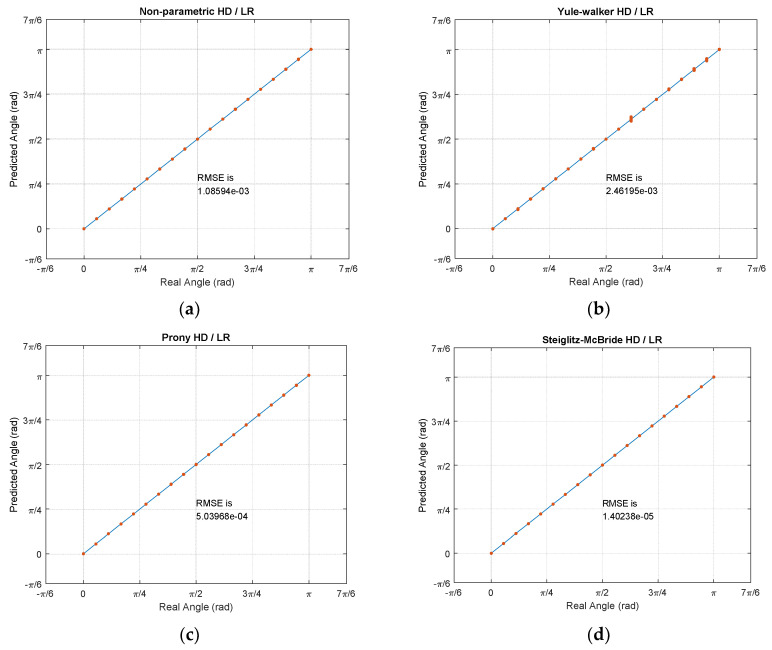
Prediction experiments with test set. (**a**) Non-parametric HD/LR; (**b**) Yule–Walker HD/LR; (**c**) Prony HD/LR; (**d**) Steiglitz–McBride HD/LR.

The actual predictions are demonstrated in Figure 15. The simulations and experiments are performed by two datasets which include the training set and test set for 1000 iterations each. The training set is annotated by the labels and test set is classified as hold-out set for blind test. All RMSEs in this paper are computed by the test set over the linear regression model which is built by training set. Note that the given frame size, overlap size, and sampling frequency provide the approximately 6.5 s for 1000 iterations. Figure 15 illustrates the individual coordinate by *x* axis with real angle and *y* axis with predicted angle. Therefore, the solid diagonal line in the figures indicates the perfect prediction. The 19 angular points are separated by π/18 radian with equiprobable chance for all iterations. The single point in the plot is the cumulated spots with highly accurate predictions. The non-parametric, Prony, and Steiglitz–McBride HD/LR show the high precision predictions with distinct points in Figure 15. The Yule–Walker HD/LR presents the spread distribution for certain angular points; therefore, the Yule–Walker HD/LR produces the least performance.

The combined RMSE distribution derives the optimal parameters for all HDs with LR. In order to reduce the bias of certain outcome, the weighted RMSE distributions are averaged for final result. The minimum RMSE of each HD/LR is normalized to the non-parametric HD/LR minimum by constant weight for individual algorithm output. The weighted HD/LR average RMSE distribution indicates the optimal parameters as ensemble length 200 and model order 14. With derived values, Table 5 demonstrates the minimum RMSE values for all HD/LR algorithms. Observe that no model order is applied to the non-parametric HD/LR algorithm. The Steiglitz–McBride HD/LR provides the best performance followed by non-parametric, Prony, and Yule–Walker HD/LR. Except the non-parametric HD/LR, the parametric HD/LRs deliver the minor performance degradation due to the quantity shift for length and order. The highest performance glitch is the Prony HD/LR RMSE with 5.2 times lower than own minimum.

**Figure 16:**
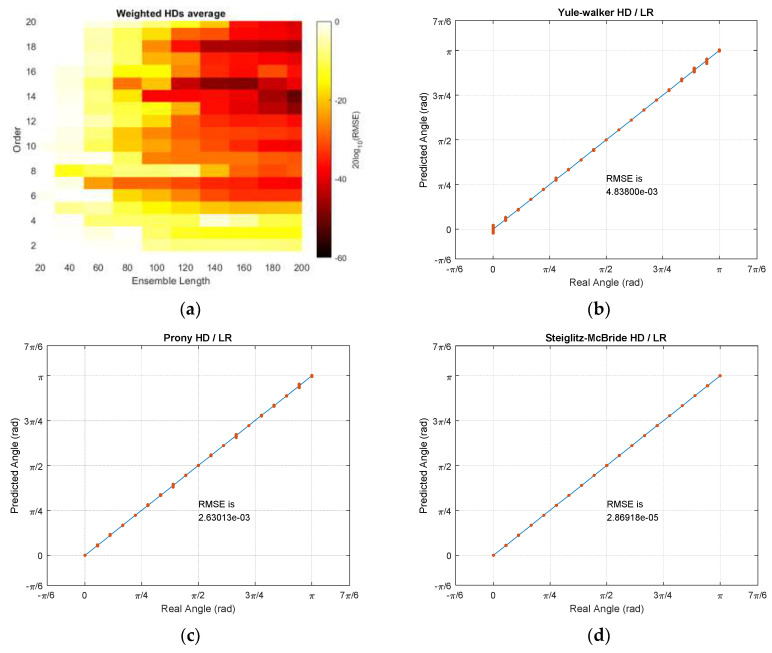
Weighted average HD/LR RMSE distribution and individual prediction plots for ensemble 200 and order 14. (**a**) Weighted average HD/LR RMSE distribution; (**b**) Yule–Walker HD/LR for ensemble 200 and order 14; (**c**) Prony HD/LR for ensemble 200 and order 14; (**d**) Steiglitz–McBride HD/LR for ensemble 200 and order 14.

The weighted average HD/LR RMSE distribution and individual prediction plots are shown in Figure 16. The average RMSE distribution presents the precise accuracy on longer ensemble length and higher model order in general. Especially, the model order 14, 15, and 18 provide the darkest shade in performance gradation on Figure 16a. The Yule–Walker and Prony HD/LR estimate the individual angle with minor variance in certain points as shown in prediction plots in Figure 16b,c, respectively. The Steiglitz–McBride HD/LR still produces very accurate prediction in Figure 16d. The non-parametric HD/LR prediction is identical to the Figure 15a since the ensemble length is 200 frames. Note that the worst minimum RMSE in Table 5 and Figure 16 is 0.2772 degree. 

The experiments in the anechoic chamber demonstrate the performance of non-parametric and parametric HDs with LR. The designed angles with π/18 apart are blindly provided to estimate the AoA with individual HD algorithms with LR. Overall, the accurate predictions are expected in proper ensemble length and model order. The optimal parameter values for each HD/LR produce the pinpoint accuracy for prediction in the descending performance order of Steiglitz–McBride, Prony, non-parametric, and Yule–Walker HD/LR. The weighted RMSE distribution derives the overall RMSE minimum at ensemble length 200 and model order 14. The obtained parameter value generates accurate estimations with minor variance. The performance can be sorted in the descending order as Steiglitz–McBride, non-parametric, Prony, and Yule–Walker HD/LR. The non-parametric HD/LR presents the consistent performance and Yule–Walker HD/LR indicates the least accuracy in simulation as well as experiments. The worst minimum RMSE in simulation and experiments are 1.2514 and 0.2772 degree, respectively. Hence, the proposed HD/LR algorithms deliver the highly accurate predictions in overall.

## 6. Conclusions

This paper presents the novel method to localize the angle of arrival by using the deconvolution with linear regression. The deconvolution is realized by the homomorphic systems in cascade to remove the sound source and to derive the propagation function which represents time of flight between the receivers. The numerical distribution of homomorphic deconvolution represents the non-parametric output for propagation. The propagation model realizes the parametric estimation by applying Yule–Walker, Prony, and Steiglitz–McBride in last stage of homomorphic deconvolution. The linear regression with terms and products is necessary to calculate the coefficients by using the feature data and QR decomposition. Once the learning process is finalized, the linear regression provides the estimated angle for the given feature input. The non-parametric and parametric homomorphic deconvolutions prepare the feature by direct feature extraction and radial projection, respectively. Among the circular configurations of receiver positions, optimal location is selected for three-receiver structure based on the extensive simulations. The simulations represent that the longer ensemble length and particular model order provide the least root mean square error. The Steiglitz–McBride shows the best performance and Yule–Walker demonstrates the least accuracy. The experiments in the anechoic chamber demonstrate the accurate predictions in proper ensemble length and model order. The non-parametric method presents the consistent performance and Yule–Walker algorithm indicates the least accuracy in simulation as well as experiments. The Steiglitz–McBride algorithm delivers the best predictions with reduced model order as well as other parameter values.

This paper extends the non-parametric and parametric homomorphic deconvolutions of previous article to the comprehensive sound localization system by linear regression. The benefits of the system include the reduced system complexity because of the single analog microphone network and the single analog-to-digital converter for multiple receivers. The second advantage of the proposition contains the absent of signal propagation model to derive the specific estimation algorithms. The training process of the linear regression provides the proper sound localization. Of course, the linear regression could involve the receiver relocations for optimal performance. Another improvement of this paper comprises the consistent algorithm complexity for receiver number. Once the ensemble length and model order are selected, the computational requirement is invariant to the sensor number. By statistical simulations and experiments, the sound localization functionality is validated for the algorithms based on the homomorphic deconvolutions with linear regression. This paper contributes to design the feasible and deployable sound localization algorithm for mobile systems by improving the scalability and complexity of the proposition. The safety of the mobile system is considerably enhanced by placing the localization receivers on any places in the system without sacrificing hardware and algorithm complexity for estimating sound arrival direction. The algorithm performance is expected to be improved by the discrete perspective from advanced learning algorithms. The massive evaluations from simulations and experiments are involved to achieve the further robust sound localization system. Future work may aim to improve the proposed localization system in sophisticated situations for instance fault-tolerance to receiver failure and middle angle predictions etc. Additionally, the paper will investigate the localization performance in the field data collected by the mobile robot with a dedicated operating system. The application of the proposed method is not limited to the sound localization because of expandability and flexibility in deconvolution and machine learning. Any sequential signals can be applied to the proposed algorithm for extraction by deconvolution and prediction by learning. The future works will help to discover the further applications other than sound localization with wide open possibilities. 

## Figures and Tables

**Table 1:** Simulation parameters and values.

Parameter	Value	Parameter	Value
Sampling frequency	48,000 Hz	Angle range	0 ~ π
Frame length	1024 samples	Angle resolution	π/35 rad (5.1429°)
Overlap length	768 samples (210–28)	Number of angles	36 angles
Ensemble length	50 frames	HD window length	5 samples
Iterations	1000 times	Max time delay (nmax)	70 samples
SNR	20 dB	Sound speed	34,613 cm/sec
Number of receivers	3	Parametric order	10

**Table 2:** Minimum RMSE values for HD/LR simulations and corresponding parameters.

Method	Non-Parametric	Yule–Walker	Prony	Steiglitz–McBride
Min. RMSE (radian)	2.1620 × 10^−3^	2.1841 × 10^−2^	2.0359 × 10^−2^	2.9541 × 10^−4^
Ensemble length (Frames)	200	200	180	160
Order	Not applicable	11	6	10

**Table 3:** Experiment parameters and values.

Parameter	Value	Parameter	Value
Sampling frequency	48,000 Hz	Angle range	0 ~ π
Frame length	1024 samples	Angle resolution	π/18 rad (10°)
Overlap length	768 samples (2^10^–2^8^)	Number of angles	19 angles
Ensemble length	Variable	HD window length	5 samples
Iterations	1000 times	Max time delay (*n_max_*)	70 samples
SNR	Not applicable	Sound speed	34,613 cm/s
Number of receivers	3	Model order	Variable

**Table 4:** Minimum RMSE values for HD/LR experiments and corresponding parameters.

Method	Non-Parametric	Yule–Walker	Prony	Steiglitz–McBride
Min. RMSE (radian)	1.0859 × 10^−3^	2.4620 × 10^−3^	5.0397 × 10^−4^	1.4024 × 10^−5^
Ensemble length (Frames)	200	200	140	200
Order	Not applicable	16	18	3

**Table 5:** Min. RMSEs for HD/LRs with single optimal parameters as ensemble 200 and order 14.

Method	Non-Parametric	Yule–Walker	Prony	Steiglitz–McBride
Min. overall RMSE (radian)	1.0859 × 10^−3^	4.8380 × 10^−3^	2.6301 × 10^−3^	2.8692 × 10^−5^

## Abbreviations

The following abbreviations are used in this manuscript (listed by order of appearance in the text).

SSL	Sound source localization
AoA	Angle of arrival
3D	Three-dimensional
NLOS	Non-line-of-sight
ADC	Analog-to-digital converter
ToF	Time of flight
HD	Homomorphic deconvolution
SCMR	Single-channel multiple-receiver
SNR	Signal-to-noise ratio
FILF	Frequency-invariant linear filtering
DFT	Discrete Fourier transform
FFT	Fast Fourier transform
RMSE	Root mean square error
LR	Linear regression
PLA	Polylactic acid
ASIO	Audio stream input/output
